# Extended-spectrum beta-lactamases: definition, history, an update on their genetic environment and detection methods

**DOI:** 10.1099/jmm.0.002033

**Published:** 2025-06-24

**Authors:** Soufyane Yassara, Ikrame Zeouk, Samira Jaouhar, Mohammed Sbiti, Khadija Bekhti

**Affiliations:** 1 Laboratory of Microbial Biotechnology & Bioactive Molecules, Faculty of Science and Technology, Sidi Mohamed Ben Abdellah University, Fez, Morocco; 2 Laboratory of Drug Sciences, Faculty of Medicine, Pharmacy and Dentistry, Sidi Mohamed Ben Abdellah University, Fez, Morocco; 3 Higher Institute of Nursing and Health Professions of Fez, Regional Directorate of Health, Meknes, Morocco; 4 Laboratory of Health Sciences and Technologies, Higher Institute of Health Sciences, Hassan First University, Settat, Morocco; 5 Microbiology Department, Moulay Ismaïl Military Hospital, Meknes, Morocco; 6 Faculty of Medicine and Pharmacy, Sidi Mohamed Ben Abdellah University, Fez, Morocco

**Keywords:** detection methods, *Enterobacteriaceae*, extended-spectrum beta-lactamase (ESBLs), genetic environment, historical evolution

## Abstract

Bacterial resistance remains a major challenge in the therapeutic field. Beta-lactam antibiotics are widely used to treat *Enterobacteriaceae*, especially third-generation cephalosporins (3GCs), which are used in infections caused by bacteria resistant to first- and second-line antibiotics. However, these bacteria have been able to develop resistance against the used antibiotics through the production of extended-spectrum beta-lactamase (ESBL) enzymes. These enzymes inactivate 3GCs and are sensitive to beta-lactamase inhibitors such as clavulanic acid. This resistance is acquired by plasmids (IncF, IncI, IncK…) which carry mobile genetic elements (insertion sequence, transposon…) with genes coding for these enzymes, namely, the *bla*_CTX-M_, *bla*_SHV_ and *bla*_TEM_, which code for the most frequent types of ESBL (CTX-M, SHV and TEM). Unfortunately, when ESBLs are not identified in time, appropriate treatment is delayed, reducing the chances of cure. Current data highlight the spread and dangerousness of ESBL-producing bacteria worldwide and confirm the priority given to these bacteria by the World Health Organization, which insists on vigilance in identifying them, both in patients and through surveillance studies. The aim of the current review is to provide a better understanding of ESBLs, to highlight their historical evolution and to show the importance of their genetic environment in the dissemination and spread of these enzymes worldwide, as well as the techniques used to detect them in laboratory studies. Current data demonstrate the degree of danger posed by ESBL-producing bacteria and confirm the priority given to these bacteria by the World Health Organization for the development of new antimicrobial agents.

## Introduction

Bacterial resistance to antimicrobial agents and its implications continues to increase, which is a major threat to human, animal and environmental health, as well as global economic development [1]. In the twenty-first century, approximately two-thirds of recent hospital prescriptions include antibiotics, particularly those belonging to the beta-lactam class (penicillins, cephalosporins, carbapenems and monobactams) [2]. The overuse of these antibiotics, particularly for the treatment of infections caused by *Enterobacteriaceae*, is leading to an increase in resistance even to antibiotics considered by the World Health Organization (WHO) to be of last resort, such as third-generation cephalosporins (3GCs) and carbapenems. This resistance is mainly caused by the production of extended-spectrum beta-lactamases (ESBLs) and carbapenemase enzymes, which are able to hydrolyze the beta-lactam ring of these antibiotics [3]. These enzymes are mainly produced by *Enterobacteriaceae*, which are classified by the WHO as priority pathogens for the research and development of new antibiotics [4]. In addition, they are resistant to a broad range of *β*-lactam antibiotics including 3GCs, penicillins and monobactams. However, this group of bacteria is inhibited by *β*-lactamase inhibitors such as sulbactam, clavulanate and tazobactam [5].

ESBL production was first reported in Germany, in 1985, by a strain of *Klebsiella ozaenae*, resistant to cefotaxime through the production of the SHV-2 enzyme derived from SHV-1 [6]. The number of ESBLs has increased over the years with the appearance of new derivatives of SHV and TEM enzymes, as well as new types of ESBL such as CTX-M, PER, GES, VEB, BES, BEL and SFO, but the most common enzymes conferring resistance to 3GC are mainly CTX, TEM and SHV derivatives [2]. The CTX-M type is the most frequent of all ESBL types, and up to now, 274 CTX-M enzymes have been identified and registered in the ‘BLDB’ database. These enzymes are mainly produced by two species belonging to the *Enterobacteriaceae* family, *Escherichia coli* and *Klebsiella pneumoniae*, which are frequently isolated from urinary tract infections and are resistant especially to ciprofloxacin [7]. The danger of these enzymes is not limited to their ability to confer resistance against a wide range of last-resort antibiotics, but also to their rapid spread, particularly for CTX-M [8], between different bacterial populations from different hosts and environments, confirming that ESBL-producing bacteria represent a major challenge in the medical field [9].

ESBL-producing *Enterobacteriaceae* represent a worldwide problem, because of their global spread in Africa, Asia, Europe, Latin America, the Middle East and North America [10]. The *bla*_CTX-M_, *bla*_SHV_ and *bla*_TEM_ genes encode the predominant ESBLs found in enterobacteria. These genes are carried by mobile genetic elements (MGEs) that may move across different bacteria, such as integrons, transposons and insertion sequences (ISs) [11]. These plasmids can carry several genes conferring resistance to other families of antibiotics such as tetracycline, sulphonamides, trimethoprim-sulphamethoxazole and fosfomycin, which makes bacteria carrying these plasmids more resistant [12].

This review aims on one hand to clarify the challenges posed by ESBL-producing bacteria, through a historical analysis of the evolution and prevalence of ESBLs, as well as their various definitions and classifications. On the other hand, it aims to show the importance of the genetic environment of the genes encoding ESBLs, and its role in the dissemination and propagation of ESBLs worldwide, and finally to highlight the different microbiological, molecular and rapid techniques for the detection of ESBLs.

## History and origin of ESBLs

The production of *β*-lactamase enzymes was observed even before penicillin entered clinical use. On 28 December 1940, Abraham and Chain identified a chromosomally encoded penicillinase capable of hydrolyzing penicillin [13]. This was attributed to environmental interactions between penicillin-producing organisms and pathogenic bacteria, especially in soil. Later, the emergence of a plasmid-encoded penicillinase, PC1, in *Staphylococcus aureus* – encoded by the *blaZ* gene – led to the rapid spread of resistance amongst Gram-positive bacteria [14]. Overuse of penicillin and dissemination of *blaZ* are largely responsible for this resistance. The percentage of penicillinase-producing *S. aureus* increased from 14% in 1946 to 80% by 1953 [15,16], and up to 86% of isolates were found to carry the *blaZ* gene [17]. In 1983, a *Streptococcus faecalis* strain (HH22) acquired penicillin resistance through a conjugation-transferable penicillinase, confirming the potential for interspecies gene transfer [18].

In many genera of Gram-negative bacteria, the production of *β*-lactamase enzymes is chromosomally mediated, but through the transmission of plasmids, the resistance began to spread widely. The first plasmid-mediated *β*-lactamase detected in Gram-negative bacteria is TEM-1 (Table 1) [19], which is involved in more than 90% of ampicillin resistance in *E. coli* and able to hydrolyze penicillin together with the first generation of cephalosporin [20]. This enzyme was first identified in the 1960s in a Greek patient named Temoniera, which explains the designation TEM [21]. Subsequently, TEM-1 was found in several species, including *Pseudomonas aeruginosa*, *Haemophilus influenzae* and *Neisseria gonorrhoeae* [22]. Then, a single amino acid substitution led to the appearance of its first variant, TEM-2 [23]. Around the same time, SHV-1 – a similar enzyme – was identified, particularly in *K. pneumoniae* [24].

**Table 1.** Examples of the first discovered beta-lactamases

*β*-Lactamase enzymes	Producing micro-organism	Year of isolation (I) or report (R)	Country	References
Penicillinases (chromosomal AmpC)	*E. coli*	1940 (I)	England	[13]
Penicillinase (plasmid-mediated PC1)	*S. aureus*	1942 (I)	England	[14]
OXA	*Salmonella typhimurium* and	1962 (I)	England	[85]
	*E. coli*	1967 (R)		[86]
TEM-1	*E. coli*	1963 (I)	Greece	[19]
SHV-1	*K. pneumoniae*	1972 (I)	Unknown	[87]
Penicillinase (class A, group 2f)	*Serratia marcescens*	1982 (I)	England	[88]
Plasmid-encoded AmpC (MIR-1)	*K. pneumoniae*	1988 (I)	USA	[89]
Plasmid-encoded MBL (IMP-1)	*P. aeruginosa*	1988 (I)	Japan	[90]

**Fig. 1.**
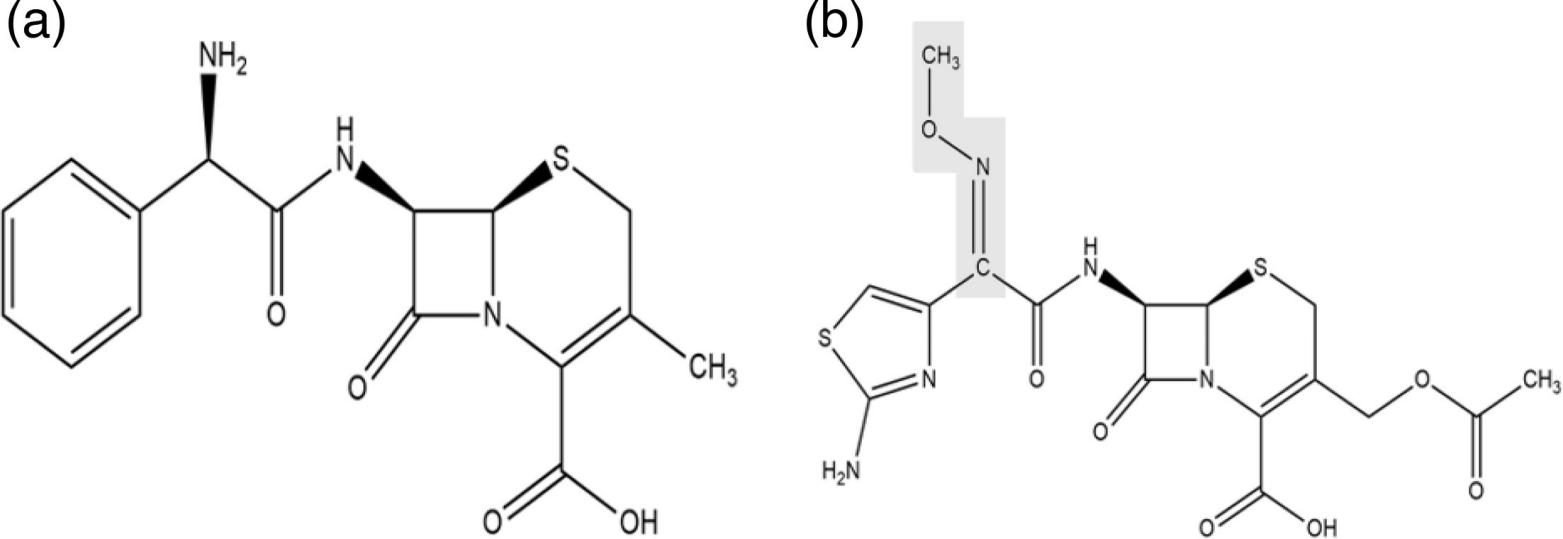
Comparison of the chemical structures of first-generation cephalosporins and oxyimino-cephalosporins. (**a**) Cefalexin and (**b**) cefotaxime. The embossed part represents the C=N OR group that protects the beta-lactam ring from attack by classical beta-lactamases, but not by extended-spectrum ones. Cefuroxime, cefotaxime, ceftriaxone, ceftazidime, cefepime and cefpirome are all designed on this scaffold.

To address *β*-lactam resistance, 3GCs – such as cefotaxime, cefuroxime, ceftriaxone, ceftazidime and cefixime – (Fig. 1) were developed in the mid-1970s to treat infections by *β*-lactamase-producing bacteria. However, extensive use of 3GCs quickly led to resistance by the early 1980s (Table 2). Kliebe *et al.* [6] reported the first *K. ozaenae* strain producing a novel *β*-lactamase, SHV-2, capable of hydrolyzing cefotaxime [25].

**Table 2.** First ESBLs

ESBL	pI	Producing micro-organism	Year of isolation (I) or report (R)	Country	References
**SHV-2**	7.6	*K. ozaenae*	1983 (I)	Germany	[6]
**CTX-1 (TEM-3**)	6.3	*K. pneumoniae*	1984 (I)	French	[26]
**SHV-3**	7.0	*K. pneumoniae*	1986 (I)	French	[28]
**TEM-4**	5.9	*E. coli*	1986 (I)	French	[91]
**TEM-6**	5.9	*E. coli*	1987 (R)	Germany	[92]
**RHH-1 (TEM-9**)	5.5	*K. pneumoniae*	1987 (R)	England	[93]
**CAZ-1 (TEM-5**)	5.55	*K. pneumoniae*	1987 (I)	French	[94]
**CAZ-2**	5.9	*K. pneumoniae*	1987 (I)	French	[95]
**CAZ-3**	5.2	*K. pneumoniae*	1987 (I)	French	[96]
**SHV-4**	7.75	*K. pneumoniae*	1987 (I)	France	[97]
**SHV-5**	8.2	*K. pneumoniae*	1987 (I)	Chile	[98]
**TEM-7**	5.41	*Citrobacter freundii*	1988 (R)	France	[99]

Around the same time, other SHV-1 mutants (SHV-4, SHV-5 and SHV-7) were found, often in nosocomial *K. pneumoniae* strains. These variants showed increased resistance to ceftazidime and aztreonam, with similar resistance to cefotaxime as SHV-2 and SHV-3 [15].

In 1987, researchers in France identified a *K. pneumoniae* strain resistant to 3GCs due to a cephalosporinase initially named CTX-1, later reclassified as TEM-3 due to its three-amino-acid difference from TEM-1 [26,27]. Frequent mutations in TEM enzymes led to many variants (TEM-4 through TEM-27), several of which share the critical substitution at position 238, associated with the intensive use of cefotaxime and ceftazidime [15].

By 1988, the modified TEM and SHV enzymes were classified as ‘extended broad-spectrum β-lactamases’ (ESBLs) for their expanded activity compared to classical Group III TEM and SHV enzymes [28]. In 1989, the term was shortened to ‘extended-spectrum β-lactamases’ (ESBLs) [29].

Whilst TEM and SHV remain prominent, CTX-M enzymes represent a significant development. First reported in 1988 in Japan by Matsumoto *et al.*, the FEC-1 enzyme from *E. coli* conferred resistance to cefuroxime, cefotaxime, cefmenoxime and ceftriaxone [30]. The following year, CTX-M-1 was identified in Germany [31], and MEN-1, an unrelated ESBL, was found in France [32,33]. MEN-1 marked the first transferable ESBL not derived from TEM or SHV. Later, Ishii *et al*. described Toho-1, sharing 83% similarity with MEN-1 [34], whilst CTX-M-2 showed 84% identity with CTX-M-1/MEN-1 [35]. In 1996, CTX-M-3 was found in cefotaxime-resistant *C. freundii* and *E. coli* in Poland [36]. FEC-1 was shown to differ from CTX-M-3 by only two signal peptide substitutions.

Additional ESBLs emerged in the late 1980s and early 1990s. In 1991, researchers in Ankara, Turkey, identified OXA-11 and OXA-14, two *P. aeruginosa* enzymes derived from OXA-10 and conferring high resistance to ceftazidime [37,38]. PER-1, first detected in Paris, also spread to Turkey, conferring resistance in *P. aeruginosa*, *Acinetobacter* and *S. typhimurium* [39,40]. PER-2 was later found in Argentina [41], whilst VEB-1 was identified in a Vietnamese patient with *E. coli* and in a Thai patient with *P. aeruginosa* [42,43]. CME-1, from *Chryseobacterium meningosepticum*, and TLA-1, from *E. coli* in Mexico, conferred broad resistance to *β*-lactams and fluoroquinolones [44,45].

The enzyme SFO-1, reported in Japan and produced by *Enterobacter cloacae*, hydrolyzes cefotaxime and shares 96% similarity with a *Serratia fonticola β*-lactamase [46]. In 1998, a *K. pneumoniae* strain (ORI-1) isolated in French Guiana produced GES-1, conferring resistance primarily to ceftazidime [47].

Looking at the historical evolution and the dissemination of ESBLs, we can note that the bacteria producing these enzymes are considered amongst the most pathogenic bacteria and present a serious problem to public health. The dissemination and the number of beta-lactamase enzymes are growing rapidly, and until the moment of writing these lines, the total number of characterized beta-lactamases registered in the database ‘BLDB’ is 11,534 of which the number of CTX-M, SHV and TEM is 286, 265 and 270, respectively [48].

## Classification

‘Extended-spectrum beta-lactamases’ represent a group of enzymes that are able to hydrolyze oxyimino-cephalosporins and are sensitive to clavulanic acid. They are classified in class 2be according to the Karen Bush classification in 1995 as clavulanate-inhibited broad-spectrum beta-lactamases capable of hydrolyzing oxyimino-cephalosporin at rates of at least 10% of that for benzylpenicillin [49]. This definition excludes other beta-lactamases with wide spectrum such as PER, CTX-M and VE13 groups, which have a different evolutionary history; some TEM mutants, which do not hydrolyse an individual oxyimino-cephalosporin at a rate equal to or greater than 10% of that of benzylpenicillin; clavulanate-resistant class C and D beta-lactamases with extended-spectrum; and some GES, which slowly hydrolyze carbapenems [50]. For these reasons, Giske *et al.* have proposed a novel beta-lactamase classification [51]. They proposed that class 2be beta-lactamases could be designated ‘class A ESBL’ (ESBL_A_), whilst plasmid-mediated AmpC and OXA-ESBL could be described as ‘miscellaneous ESBL’ (ESBL_M_). Then, this category might be divided into two classes: ESBL_M-C_ (plasmid-mediated AmpC; class C) and ESBL_M-D_ (OXA-ESBLs; class D). For carbapenemases, only enzymes with an imipenemase activity (k_cat_/V_max_) of >1 (µM^−1^s^−1^) are designated as ESBL_CARBA_ (carbapenem-hydrolyzing ESBL), and further details are summarized in Table 3.

**Table 3.** Proposed classification of ESBL_A_, ESBL_M_ and ESBL_CARBA_ [51]

	ESBL_A_		ESBL_M_		ESBL_CARBA_		
	**High-prevalence ESBL_A_**	**Low-prevalence ESBL_A_**	**ESBL_M-C_ (plasmid-mediated AmpC**)	**ESBL_M-D_ (OXA-ESBL**)	**ESBL_CARBA-A_**	**ESBL_CARBA-B_ (MBL**)	**ESBL_CARBA-D_ (OXA-carbapenemases**)
Beta-lactamase classes	CTX-M, TEM-ESBLs, SHV-ESBLs, VEB and PER	GES-1,-3,-7,-9, SFO-1, BES-1, BEL-1, TLA, IBC and CMT*	CMY, FOX, MIR, MOX, DHA, LAT, BIL, ACT and ACC	OXA-10 group, OXA-13 group, OXA-2 group, OXA-18 and OXA-45	KPC, GES-2,-4,-5,-6,-8 NMC, SME and IMI-1,-2	IMP, VIM, SPM-1, GIM-1, SIM-1 and AIM-1	OXA-23 group, OXA-24 group, OXA-48† and OXA-58 group
Operational definition	Non-susceptibility to extended-spectrum cephalosporins AND clavulanate synergy		Non-susceptibility to extended-spectrum cephalosporins AND phenotypic detection (ESBL_M-C_) OR genotypic detection (ESBL_M-D_)		Non-susceptibility to extended-spectrum cephalosporins and at least one carbapenem AND ESBL_CARBA_ detected with phenotypic and/or genotypic methods		

*Resistant to clavulanic acid inhibition.

†OXA-48-producing isolates may appear susceptible to cephalosporins *in vitro*.

## Plasmids and the genetic environment of ESBL genes

**Fig. 2.**
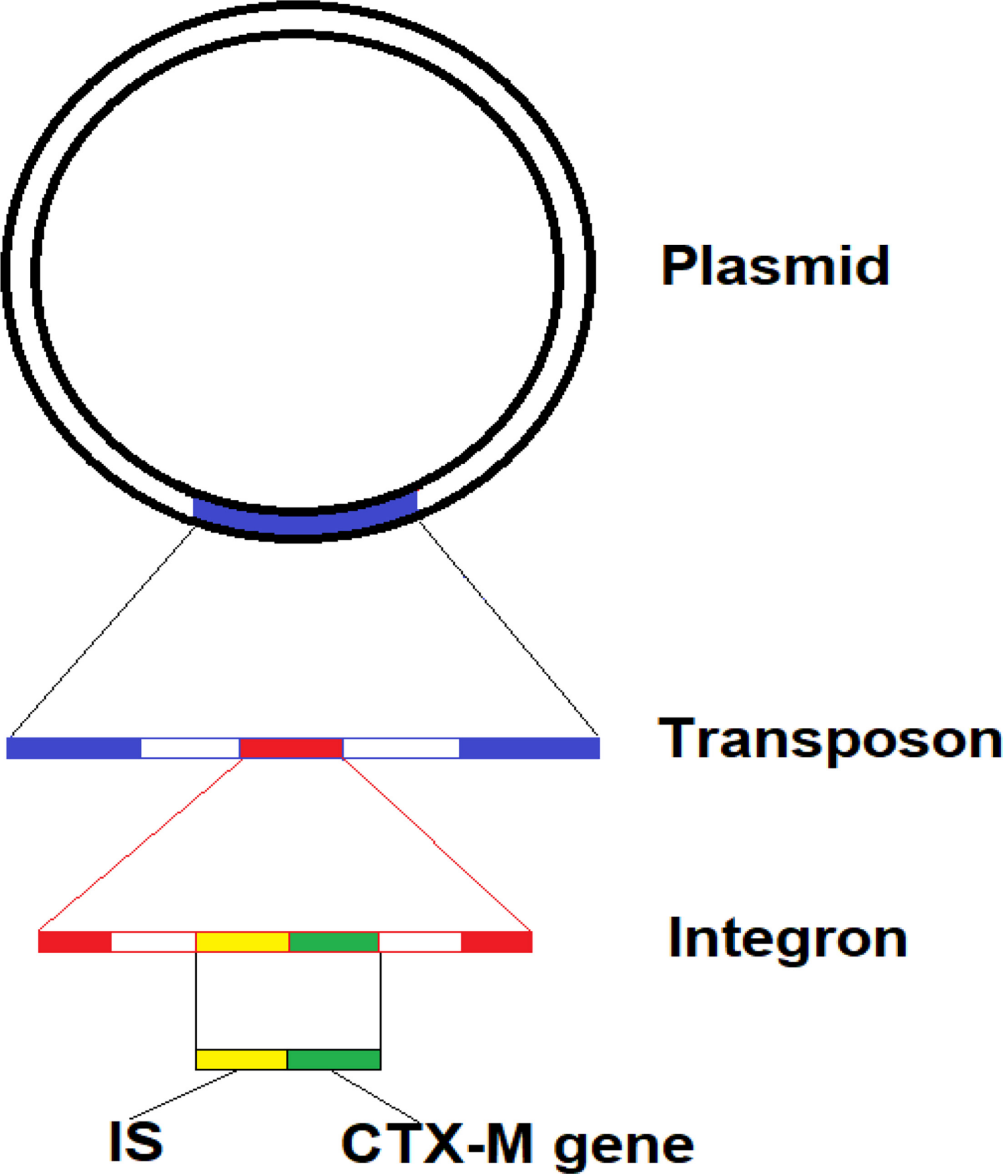
Model of a plasmid carrying a CTX-M gene in an integron. Plasmids can carry transposons, which facilitate the dissemination of resistance genes and are characterized by the presence of integrons carrying ISs.

Enterobacterial resistance to 3GCs has become more complex due to the emergence of bacterial clones and the dissemination of ESBL-encoding genes by MGEs such as plasmids. These may contain ISs, transposons and integrons which can move from one place to another within the bacterium or be transferred from one bacterium to another horizontally by conjugation and transformation or, in the case of bacteriophages, by transduction [52]. In some cases, the ISs adjacent to the *bla*_CTX_ gene are integrated into integrons, which are also part of transposons (Fig. 2).

The spread of plasmids carrying resistance genes within different bacterial species results from the pressure exerted by the application of new extended-spectrum antibiotics. There are several families of plasmids carrying ESBL-encoding genes, as well as resistance genes for other antimicrobial classes. For example, in GenBank, the fully sequenced plasmid families containing *bla*_CTX-M-15_ genes found in *E. coli* are IncF, IncN, IncN2, IncI1, IncHI2, IncL/M, IncA/C, IncK, IncX4, IncU and RCR, of which the IncF family is most responsible for ESBL production in *E. coli* [53,54].

Many studies have been carried out to demonstrate the importance of plasmids in the dissemination of ESBL-encoding genes (Table 4). A study carried out on a total of 113 plasmids showed 64 of 82 conjugative plasmids carrying ESBL-encoding genes (CTX-M, TEM and SHV), whereas plasmids carried several genes belonging to the IncF, IncI1, IncA/C and IncL/M families. Seven IncF plasmids carrying the *bla*_CTX-M-15_ gene inserted in a Tn*2-bla*TEM-1 transposon, with many other genes (CTX-M and SHV), confirm that this family of plasmids plays a very important role in resistance diffusion [55]. IncF plasmids are highly diverse in terms of plasmid size, number of replicons and conjugation capacity. They are generally large in size and contain virulence and resistance genes that are mostly *bla*_CTX-M-15_ [56]. ESBL-producing *E. coli* ST131 is the most virulent and dominant strain worldwide. It is associated with IncF plasmids, which demonstrate that this plasmid plays a key role in the dissemination of ESBL genes, especially *bla*_CTX-M_ [57]. In East Africa, IncF plasmids such as IncFIA, IncFIB, IncFII and IncFIB (K) have been reported to be associated with *E. coli*-producing ESBL collected from animals, humans and the environment. Similar associations have also been observed in ESBL-carrying isolates of *K. pneumoniae* and *E. cloacae* [58].

**Table 4.** ESBL-encoding plasmids and their genetic environment

Plasmids (accession no.)	Size (pb)	Family	GC%	ESBL-resistance genes	Other resistance genes	MGEs	Micro-organisms	References
pMGRU2 5-002 (KT818627)	172,679	–	52.6	*bla*_CTX-M-15_, *bla*_SHV-1 1_, *bla*_TEM-1A,1B_ and *bla*_OXA-9_	*strAB*, *aadA1*, *aac(6’)-Ib*, *aac(3)-IId*, *sul1*, *sul2*, *cmlA1*, *erm(B*) and *mph(A*)	–	*K. pneumoniae* U25	[100]
pKp_Goe _208-2 (CP01844 9)	67,101	IncL/M	–	*bla*_CTX-M-14b_ and *bla*_OXA-48_	*qnrS1*, *qnrS9*, *aph(6)-Id*, *aph(30)-VI* and *aph(3’*)	Tn*1999.2*, IS*6*, IS*1* and IS*3* families	*K. pneumoniae* Kp_Goe_33208	[101]
pKp_Goe _641-1 (CP018737)	72,952	IncR	–	*bla*_CTX-M-15_, *bla*_TEM-1A_ and *bla*_OXA-9_	*bla*_OXA-1_, *ant(3’)-Ia*, *aac(60)-Ib*, *aac(60)-Ib-cr*, *aac(3)-IIa*, *dfrA14*, *cat*, *merE*, *merT* and *merC*	IS*6*, IS*1* (IS*1* family IS*Ecp1* element), Tn*21*, Tn*3*, IS*256* and IS*3*	*K. pneumoniae* Kp_Goe_121641	
pKp_Goe _414-6 (CP018343)	57,226	IncF (FII)	–	*bla*_CTX-M-55_	*bla*_OXA-1_, *aac(3)-IIa*, *aac(60)-Ib-crc* and *atB3*	Tn*3*, IS*6*, IS66, IS*1*, Tn*1721* (Tn*3*) and IS*3*	*K. pneumoniae* Kp_Goe_154414	
pKp_Goe _795-1 (CP018460)	232,181	IncF(FIB)	–	*bla*_CTX-M-15_ and *bla*_TEM-1B_	*bla*_OXA-1_, *aac(60)-Ib-cr*, *aac(3)-IIa*, *aph(6)-Id*, *aph(3’)-Ib*, *ant(3’)-Ia*, *catB3, catA1*, *sul2*, *tetA*, *tetR* and heavy metal (arsenic, copper, cobalt, zinc and cadmium) resistance determinants	IS*6*, Tn*3*, Tn*p1* (IS*1380* le), IS*110* le, *IntIPac*, IS*1*, IS*NCY* le, IS*L3*, IS*66*, IS*3*, IS*481* le and IS*903*	*K. pneumoniae* Kp_Goe_39795	
pKp_Goe_917-1 (CP018441)	180,027	IncF (FII+FIB)	–	*bla*_CTX-M-15_	*bla*_OXA-1_, *aac(60)-Ib-cr*, *aac(3)-IIa*, *aph(30)-Ia*, *aac(30)-III catB3*, *dfrA14* and heavy metal (arsenic, copper, cobalt, zinc and cadmium) resistance determinants	IS*903*, Tn*3*, IS*6*, integron integrase *IntIpac* and IS*5* (IS*1182-DUF772*)	*K. pneumoniae* Kp_Goe_822917	
p48212_ MCR (CP059413)	302,551	IncHI2	–	*bla*_SHV-12_ and *bla*_TEM-1b_	*mcr-9*, *aac(69)-IIc*, *aadA2b*, *aph(6)-Id*, *dfrA19*, *catA2*, *sul1*, *sul2*, *tetD*, *aac(69)-Ib-cr*, *qnrA1* and *ere(A*)	–	*Enterobacter hormaechei* ST106 ENCL48212	[102]
p16005813C (MK0368 84)	61,463	IncR	53.2	*bla*_CTX-M-9_	*mer* locus, *catA1*, *catB8*, *sul1*, *aacA4* and *tet(C*)	IS*CR1*, IS*26*, IS*1R*, IS*4321R*, Tn*6322*, Tn*9*, Tn*21* and In609	*Leclercia adecarboxylata* 16005813	[103]
pKOX752 5_1 (CP065475)	397,447	IncHI5B, IncFIA and IncR	49.1	*bla*_SFO-1_ and *bla*_OXA-10_	*bla*_IMP-4_, *bla*_NDM-1_, *mph(A)*, *aph(6)-Id*, *aph(3’)-Ib*, *catB3*, *qacG2*, *sul1*, *aadA16*, *dfrA27*, *arr-3*, *aadA1* and *ble*_MBL_	IS*6100*, *IS26*, *IS5075* and Tn*5393*	*Klebsiella michiganensis* 7525	[104]
pKPNT46-121	329,420	IncHII	48.24	*bla*_SHV-12_, *bla*_DHA-1_ and *bla*_TEM-1B_	*bla*_IMP-26_, *fosA5*, *qnrB4*, *aac(6′)-Ib3*, *aac(6′)-IIc*, *aacA4*, *aph(6)-Id*, *strA*, *mph(A)*, *ere(A)*, *catA2*, *tet(D)*, *dfrA18* and *sul1*	IS*4*, IS*6*, IS*26*, IS*CR1*, Tn*3* family and *intI1*	*E. cloacae* RJ702	[105]
pS1_Plas2	76,469	IncFII	–	*bla*_CTX-M-55_	*bla*_TEM_, *rmtB1*, *fosA3*, TetR and *aph(3’)-IIa*	IS*26*, *orf477*, IS*Ecp1*, IS*1* and IS*91*	*E. coli* S1	[106]
pKPN-T46-121	121,145	IncFII and IncQ1	52.3	*bla*_CTX-M-3_ and *bla*_TEM-1B_	*sul2*, *strA*, *strB*, *tet(A)*, *floR* and *qnrS1*	IS*26*, IS*Swi1*, IS*kpn24*, IS*Soen2* and IS*1×2*	*K. pneumoniae* ST23	[107]
pKW21 (CP117673)	179,817	IncF	–	*bla*_CTX-M-55_ and *bla*_TEM-1B_	*aac(30)-IIa*, *aph(30)-Ib*, *mph(A)*, *aph(60)-Id*, *tet(R)*, *tet(A)*, *floR, fosA3* and *aadA5*	IS*26*, IS*4* family, Tn*3*, Tn*2*, IS*Ecp1*, IS*1R*, IS*5075*, IS*91* and IS*Cfr1*	*E. coli* KW21	[108]
pCFSA629 (CP033351)	210,674	IncHI2	45.2	*bla*_CTX-M-14_	*fosA3*, *mcr-1*, *aac(3)-IV* and *aph(4)-Ia*	IS*30* and IS*6* families, IS*26*, IS*1182*, IS*Apl1*, Tn*As1*, Tn*6330* and Tn*3*	*Salmonella enterica* CFSA629	[109]
pKPT698-tmexCD (CP079784)	291,624	IncHI1B/FIB	46.8	*bla*_DHA-1_	*tmexCD1*, *toprJ1*, *aadA1*, *aadA2*, *cmlA1*, *sul3*, *aac(3)-IVa*, *aph(4)-Ia*, *aph(30)-Ic*, *strA*, *strB*, *mph(E)*, *msr(E)*, *armA*, *sul1*, *tet(B*) and *qnrB4*	Tn*As1*, Tn*5393*, IS*Ec28*, IS*Ec29*, IS*Ec59*, IS*903*, IS*CR1*, IS*1*, IS*26* and *IntI1*	*K. pneumoniae* T698-1	[110]
pKPT698-mcr (CP079783)	90,695	IncFII/FIA	50.1	*bla*_CTX-M-15_ and *bla*_TEM−1B_	*mcr-8.2*	Tn*2*, IS*1*, IS*26*, IS*Kpn26*, IS*903* and IS*Ecl1*		
pCFSA664-1 (CP033353)	255,327	IncHI2, IncHI2A and IncN	47.9	*bla*_CTX-M-65_, *bla*_OXA-1_ and *bla*_TEM-1B_	*mph(A)*, *aph(3’)- IIa*, *aadA5*, *aph(6)-Id*, *aph(3″)-Ib*, *aac(3)-IVa*, *aph(4)-Ia*, *aac(6′)-Ib-cr*, *tet(A)*, *fosA3*, *sul1*, *sul2*, *catB’*, *arr3*, *qacE-1*, *floR*, *dfrA17*, *aadA5*, *oqxA*, *oqxB* and *aac* (incomplete)	IS*1*, IS*3*, IS*4*, IS*5*, IS*6*, IS*91*, IS*26*, IS*50R*, IS*1006*, IS*Vsa3*, Tn*3*, Tn*AS1*, Tn*As3* and *intI1*	*S. enterica* CFSA664	[111]
pK012_2 (RSEZ01000005)	146,262	IncFII and IncR	–	*bla*_CTX-M-15_ and *bla*_TEM−1B_	*catA2*, *aac(3)-IId* and *dfrA30*	IS*26*, IS*Ecp1*, IS*5075*, IS*Vsa3*, IS*Kpn24* and IS*kpn1*	*K. pneumoniae* 12	[112]
pLV23529-CTX-M-8 (KY964068)	89,458	IncI1	–	*bla*_CTX-M-8_	*bla*_TEM−1_	IS*26*, IS*Vsa5* and Tn*pA*	*E. coli* LV23529	[113]
pDKB1765	98,488	IncY	–	*bla*_CTX-M-15_	*bla*_TEM−1_, *qnrS1*, *tet(A)*, *aph(6)-Id* and *sul2*	IS*91*, IS*1380*, Tn*pA* and Tn*3*	*E. coli* DKB1765	[114]
P1A4 (CP075623)	105,608	IncI1	50.2	*bla*_CTX-M-55_	*lnu(F)*, *aadA17* and *aac(3)-IId*	IS*3*, IS*66*, IS*26*, IS*Ec9*, Tn*3* and *intI1*	*E. coli* 1A4	[115]
p9C1 (CP075617)	99,872	IncB/O/K/Z	52.3	*bla*_CMY-2_	–	IS*Ec9*, IS*21* and IS*Ec12*	*E. coli* 9C1	
p14154A (CP064667)	202,724	IncHI2-IncHI2A	–	*bla*_CTX-M-65_	*bla*_TEM-1_, *bla*_OXA-1_, *mphA*, *strA*, *strB*, *sul1*, *sul2*, *floR*, *aadA5*, *dfrA17*, *qepA*, *oqxAB*, *fosA3*, *aph(4)-I*, *qacE*, *arr-3*, *catB*, *aac(6’)-Ib-cr*, *aadA, cmIA*, *aadA2, aac(3)-II* and *rmtB*	IS*15*, IS*4*-like, IS*91*-like, IS*Ec59*, IS*26*, IS*6100*, IS*Ecp1*, IS*CR3*, IS*Vsa3*, IS*1006*, IS*903B*, Tn*1721*, Tn*AS3*, Tn*AS1* and Tn*3*-like	*Salmonella indiana* SJTUF14154	[116]
p288_18	67,262	IncFII	–	*bla*_CTX-M-55_ and *bla*_TEM−1B_	–	IS*26*	*S. enterica* ST13	[117]
pLAO37 (OP242229)	98,237	IncFII2	–	*bla*_CTX-M-55_	*mcr-3.4*, *tmrB*, *aacC2d*, *qnrS1* and *catA2*	IS*Ecp1*, IS*26*, IS*10*, IS*Kpn26*, IS*1*, Tn*2*, Tn*21* and Tn*1721*	*E. coli* LA110	[118]
pUR5279 (OQ747075)	98,339	IncF	–	*bla*_SHV-12_	*sul3*, *aadA1*, *aadA2*, *qacL*, *aph(6)-Id* and *cmlA1*	Tn*1721*, Tn*21*, IS*26*, IS*Kpn26*, IS*Ecp1*, IS*1294* and In*2*	*E. coli* X5279	[119]
pUR5239 (OQ747074)	96,333	IncF	–	*bla*_CTX-M-32_ and *bla*_TEM−1A_	*aadA22*, *aph(3’)-Ib*, *aph(6)-Id*, *tet(A)*, *strA* and *strB*	Tn*1721*, Tn*21*, Tn*p256*-like, IS*1*, IS*26*, IS*66*, IS*Kpn26*, IS*Ecp1*, IS*1294*, IS*256* and In*2*	*E. coli* X5239	

CTX-M are the most widespread type of ESBL enzymes, and their genes are frequently disseminated between enterobacteria via IS*Ecp1* which has been identified upstream of several *bla*_CTX-M_ types [59,60]. Originally, these were chromosomal genes of *Kluyvera* spp., but they were transferred into plasmids by MGEs (IS), and thanks to mutations, there are currently several types of CTX-M. As a consequence, they have become capable of hydrolyzing ceftazidime as well as cefotaxime [61]. The dissemination of *bla*_CTX-M_ genes is not limited to IncF plasmids, but different families of plasmids have been detected.

Many studies have been carried out in African countries and have shown that in all these countries, several IncF plasmids in *E. coli* strains carry the *bla*_CTX-M-15_ genes, but they are also found to be associated with a variety of other plasmid replicons, including rare plasmid types such as IncY and IncQ [62]. A study carried out in Mwanza, Tanzania, on environmental isolates and fish viscera from the shores of Lake Victoria showed that 21 strains carry the *bla*_CTX-M-15_ gene, and genes for resistance to tetracyclines [tet(A)/tet(B)], sulphonamides (sul1/sul2) and aminoglycosides [*aac(3)-lld*, strB and strA]; fluoroquinolones [*aac(6′)-Ib-cr*, *qnrS1*]; and trimethoprim (*dfrA14*) were detected. Seven IncF plasmids were detected in fish isolates and five in environmental isolates, whilst IncY plasmids carrying *bla*_CTX-M-15_, *strA*, *qnrS1* and *strB* were detected in five environmental *E. coli* isolates and one fish *E. coli* isolate. The *bla*_CTX-M-15_ genes are located between IS*Ecp1* and transposon Tn*3* [63]. A study carried out on the faeces of travellers returning to the UK showed that 174 *E. coli* isolates, including 21 belonging to the ST131 clone, harboured the *bla*_CTX-M-15_ gene; the upstream environment of this gene consisted of intact IS*Ecp1* (*n*=108), different lengths of truncated IS*Ecp1* (*n*=58) or the remainder of 24 bp IS*Ecp1* (*n*=8), whereas *bla*_CTX-M-15_ is transcribed by two different promoters, resulting in different levels of cephalosporin resistance [64].

The second most common CTX-M ESBL is CTX-M-14 (a member of the CTX-M-9 group). It has been detected in a strain of *E. coli* isolated from cattle in the UK. This enzyme is disseminated by the pCT plasmid (GenBank accession no. F868832.1), which belongs to the IncK family. pCT-like plasmids have also been detected in *E. coli* isolates in Spain, Australia and China [65]. *E. cloacae* ST133 has been detected to harbour an IncHI2 plasmid containing *bla*_CTX-M-15_, *bla*_SHV-12_ and *bla*_NDM-1_ genes which confer resistance to carbapenems [66]. A *K. pneumoniae* isolate was detected carrying a *bla*_CTX-M-15_ ESBL gene associated with an IS*Ecp1* IS. This gene truncated the *mgrB* gene between the +21 and +22 nucleotides, resulting in the acquisition of colistin resistance [67]. In southern Chile, 137 ESBL-producing bacteria were isolated. These isolates were *K. pneumoniae* (*n*=115), *E. coli* (*n*=18), *Proteus mirabilis* (*n*=3) and *E. cloacae* (*n*=1). A large number of ESBL genes have been detected by PCR amplification in ESBL-producing isolates. The most prevalent ESBL genes were *bla*_CTX-M-1_ and *bla*_SHV_-like, present in 85% and 89% of *K. pneumoniae* isolates, respectively; *E. coli* carried *bla*_CTX-M-1_ and *bla*_TEM_ in 83% and 67% of isolates, respectively. Other genes were detected with low percentages compared to the others, which are *bla*_CTX-M-2_ (*n*=28/20.4%), *bla*_CTX-M-9_ (0.7%), *bla*_PER-1_ (0.7%) and *bla*_GES-10_ (0.7%) [68].

The IncI1 family of plasmids plays an important role in the dissemination of CTX-M-1 and CTX-M-2 beta-lactamases amongst European human and farmed *Enterobacteriaceae* isolates. An IncI1 plasmid has been detected associated with the *bla*_CTX-M-2_ gene in *E. coli* isolates from faecal samples of healthy thoroughbred racehorses in Japan. These isolates have also been carried out in *bla*_CTX-M-1_ and *bla*_TEM-116_ [69]. Numerous I1 lines have been shown to carry *bla*_CTX-M-1_, but ST3 has repeatedly been identified as the prevalent vehicle amongst both *E. coli* and *S. enterica* isolates from poultry in Europe. Also, other plasmid families such as HI2, K and N-carrying ESBL genes have also been documented in wildlife isolates [70]. The discovery of IncI1/ST3 plasmids containing *bla*_CTX-M-1_ in isolates of *K. pneumoniae* and *E. coli* raises the possibility that *bla*_CTX-M-1_ can spread horizontally between different bacterial species, considering that *E. coli* is one of the possible reservoirs for the persistence and environmental mobility of this gene [71].

## Methods of ESBL detection

ESBL detection has become a requirement for differentiating between certain strains that produce ESBL but are susceptible to cephalosporins. The frequency of ESBL-producing micro-organisms susceptible to cephalosporins varies according to several factors that can be taken into consideration. The interpretation of cephalosporin susceptibility depends on the breakpoints used in each country; for example, susceptibility to ceftazidime may be reported for an organism with MICs ranging from ≤1 to ≤8 µg ml^−1^, depending on the country, whereas resistance is from ≥2 to ≥64 µg ml^−1^ [72]. The different beta-lactamases produced and the enhanced penetration of certain cephalosporins through the bacterial outer membrane compared with others lead to different degrees of cephalosporin hydrolysis, which explains the difference in sensitivity to certain cephalosporins [29]. Inoculum concentration is also an important factor. *In vitro*, cephalosporin MICs increase with increasing inoculum [73]. As a result, MICs alone can give erroneous information. For these reasons, the Clinical and Laboratory Standards Institute (CLSI) and the European Committee on Antimicrobial Susceptibility Testing (EUCAST) have recommended several methods for ESBL detection. These methods take advantage of the fact that ESBLs are inhibited by traditional beta-lactamase inhibitors such as clavulanate but differ in the micro-organisms recommended for test, screening and confirmation tests and interpretations (Table 5).

**Table 5.** ESBL screening and confirmation tests recommended by CLSI and EUCAST [11]

Criteria	CLSI	EUCAST
**Organisms**	*E. coli*, *Klebsiella oxytoca*, *K. pneumoniae* and *P. mirabilis*	Group 1: *E. coli*, *Klebsiella* spp*.* (not including *Klebsiella aerogenes*), *P. mirabilis*, *Raoultella* spp., *Salmonella* spp. and *Shigella* spp.Group 2 (*Enterobacterales* with inducible chromosomal AmpC): *Enterobacter* spp., *C. freundii*, *Morganella morganii*, *Providencia stuartii*, *Serratia* spp. and *Hafnia alvei*
**Screening test methods**	Disc diffusion and BMD methods	Broth dilution, agar dilution or disc diffusion
**Screening agents and cutoffs**	Aztreonam, cefotaxime, ceftazidime and ceftriaxone MIC of ≥2 mg l^−1^Cefpodoxime MIC of ≥2 mg l^−1^ for *P. mirabilis* or MIC≥8 mg l^−1^ for *E. coli*, *K. pneumoniae* and *K. oxytoca*	Cefpodoxime, cefotaxime, ceftazidime and ceftriaxone MIC of ≥2 mg l^−1^
**Positive screening results**	Either (i) cefpodoxime alone or (ii) aztreonam (excluding *P. mirabilis*), cefotaxime, ceftazidime or ceftriaxone screen positive	Either (i) cefpodoxime alone or (ii) cefotaxime or ceftriaxone AND ceftazidime screen positive
**Confirmatory test methods**	Disc diffusion and BMD methods	CDT, DDST, ESBL gradient test and BMD test
**Test**	Ceftazidime and cefotaxime+clavulanate	Group 1: ceftazidime and cefotaxime+clavulanate;add cefepime+clavulanate if cefoxitin has been tested and has an MIC of ≥16 mg l^−1^Group 2: cefepime+clavulanate
**Positive interpretation**	Disc diffusion: ≥5 mm increase in zone diameter for either agent tested in combination with clavulanate versus the zone diameter of the agent tested alone.BMD: ≥3 twofold concentration decreases in an MIC for either agent tested in combination with clavulanate versus the MIC of the agent tested alone	CDT: same interpretation as the CLSI disc diffusion testDDST: zones of inhibition around cephalosporin discs are augmented, or there is a keyhole in the direction of the disc containing clavulanateBMD: ≥8-fold reduction is observed in the MIC of the cephalosporin combined with clavulanate compared with the MIC of the cephalosporin aloneGradient diffusion: the same as above for BMD or if a phantom zone or deformed ellipse is present

BMD, broth microdilution; CDT, combination disc test; DDST, double-disc synergy test.

The table shows microbiological techniques (disc diffusion, agar dilution, BMD…) for the detection of ESBLs. These techniques require overnight incubation and have known some limitations that affect both sensitivity (false negatives due to co-production of an AmpC beta-lactamase) and specificity (false positives due to hyperproduction of narrower-spectrum beta-lactamases combined with altered permeability). In order to increase the sensitivity of identification of ESBL-producing organisms, CLSI and EUCAST have decided to lower the cephalosporin breakpoints, which will not only reduce the cost of confirmatory testing for microbiology laboratories but also update pharmacokinetic/pharmacodynamic data, MIC distributions and limited clinical outcome data to improve results for patients with lower breakpoints [74,75].

In addition to these techniques, there are other sophisticated phenotypic techniques for confirming the presence of ESBLs. The E-test (or gradient strip test) is a quantitative method that uses a gradient of antibiotic alone and in combination with clavulanic acid, revealing a significant decrease in the MIC, a telltale sign of the presence of ESBLs [76]. CHROMagar ESBL is a selective chromogenic medium that simplifies the rapid identification of ESBL-producing bacteria by creating colonies of distinct colours for each species, whilst inhibiting the growth of non-producing bacteria [76]. The three-dimensional assay (direct or indirect) involves examining the deformation of inhibition zones on an agar medium due to the hydrolyzing enzymatic activity of ESBLs, by placing the inoculum in a notch close to the antibiotic disc. Finally, the modified synergy test is an advanced version of the double-disc synergy test. It uses various third- and fourth-generation cephalosporins arranged around a central disc of amoxicillin-clavulanic acid, enabling the detection of ESBLs and the distinction between AmpC producers [77].

Newer rapid phenotypic methods can also be used in microbiology laboratories, giving same-day results for the detection of ESBL-producing organisms. Firstly, a colourimetric assay gives results within 15 min to 2 h and includes methods that specifically detect ESBL producers [Rapid ESBL NDP or Rosco Diagnostica Rapid ESBL Screen (Taastrup, Denmark)] or more broadly detect ESBLs, AmpCs and carbapenemases without distinction due to cleavage of a chromogenic extended-spectrum cephalosporin (b Lacta Test; Bio-Rad, Marnes-La-Coquette, France) [78]; secondly, a lateral flow immunoassay (NG-Test CTX-M MULTI assay, NG Biotech, Guipry, France) allows detection and differentiation of the five CTX-M enzyme groups (groups 1, 2, 8, 9 and 25) from colonies and positive blood cultures in about 15 min, with excellent sensitivity and specificity (98%) [79,80].

In parallel to phenotypic methods, molecular methods that have the advantage of targeting specific ESBL genes have been developed to be used in microbiology laboratories. The syndromic sepsis panels are the most widely adopted because of their rapidity and their good sensitivity and specificity for the detection of *bla*_CTX-M_. They are based on positive blood culture broths that include CTX-M as the only ESBL target to detect *Enterobacteriaceae*, *P. aeruginosa* and/or *Acinetobacter baumannii*. This gene is the most dominant worldwide, and its detection enables more rapid selection of the appropriate therapy. These panels include the GenMark Dx ePlexVR Blood Culture Identification Gram-Negative Panel (Carlsbad, CA, USA), the BioFire BCID2 Panel (Salt Lake City, UT, USA) and the Verigene Gram-Negative Blood Culture Nucleic Acid Test BC-GN panel (Austin, TX, USA) [81]. Multiplex pneumoniae panels for direct detection from respiratory samples are also targeting the gene encoding CTX-M, such as the Unyvero LRT panel (Curetis, Holzgerlingen, Germany), which demonstrated 95.7% sensitivity for the detection of *bla*_CTX-M_, and the BioFire FilmArray pneumonia panel (Salt Lake City, UT, USA), which can reach 85.7% sensitivity [82,83]. Molecular methods have many advantages, but they also have disadvantages (Table 6).

**Table 6.** Advantages and disadvantages of molecular methods for ESBL detection [22,84]

Test	Advantages	Disadvantages
PCR	Easy to perform, specific for gene family (TEM or SHV)	Cannot distinguish between ESBLs and non-ESBLs, cannot distinguish between variants of TEM or SHV
Real-time PCR	Identify several ESBL genes, SNP detection and detection of key mutations in the SHV, TEM and CTX-M-type ESBLs	Due to the limited set of fluorescent labels, synchronic detection is possible for only a few SNPs.
DNA probes	Specific for gene family (TEM or SHV)	Labour-intensive, cannot distinguish between ESBLs and non-ESBLs, cannot distinguish between variants of TEM or SHV
Oligotyping	Detects specific TEM variants	Requires specific oligonucleotide probes, labour-intensive, cannot detect new variants
PCR single-strand conformational polymorphism	Can distinguish between a number of SHV variants	Requires special electrophoresis conditions
PCR-restriction fragment length polymorphism	Easy to perform, can detect specific nucleotide changes	Nucleotide changes must result in an altered restriction site for detection.
Ligase chain reaction	Can distinguish between a number of SHV variants	Requires a large number of oligonucleotide primers
Microarray	Detect a very large number of genes, identify SNPs from 96% of TEM variants	Labour-intensive, need specific measuring scanners
MALDI-TOF MS-based mini-sequencing method	Detect SNPs in the resistance gene and nucleotide mutations	Labour-intensive and take a lot of time
Nucleotide sequencing	The gold standard, can detect all variants	Labour-intensive, can be technically challenging, can be difficult to interpret manual methods

Genotyping is a powerful method for accurately identifying ESBL-encoding genes. Conventional PCR enables targeted amplification of genes such as *bla*_CTX-M_, *bla*\sub>TEM or *bla*\sub>SHV, whilst real-time PCR enables rapid and accurate measurement of gene expression, frequently used in clinical diagnostics. DNA probes have a high degree of specificity in detecting complementary sequences by hybridization. By analysing certain oligonucleotide sequences, oligotyping enables similar geometric variations to be differentiated. The PCR-SSCP approach distinguishes different gene variants on the basis of the conformation that the DNA adopts after amplification. To identify genetic polymorphisms by electrophoresis, PCR-RFLP combines amplification and enzymatic digestion of DNA. An alternative to PCR for the detection of sporadic mutations is the ligase chain reaction, which uses the activity of a ligase. Hundreds of resistance genes can be detected simultaneously on a single platform using DNA microarrays. MALDI-TOF MS-based mini-sequencing is used to rapidly identify ESBL mutations on the basis of a mass profile. Finally, nucleotide sequencing, also known as next-generation sequencing, is the gold standard for the fine identification of genetic variations. It enables in-depth characterization of resistance genes and their genetic context [22,84].

## Conclusion

ESBL-producing bacteria are a real threat to public health. They are frequently produced by bacteria belonging to the *Enterobacteriaceae* family, particularly *E. coli* and *K. pneumoniae*. The number and prevalence of ESBLs have been rising since their first discovery in 1985, in *K. ozaenae*. The most common ESBL types are CTX-M, SHV and TEM. They are encoded by the *bla*_CTX-M_, *bla*_SHV_ and *bla*_TEM_ genes, but the *bla*_CTX-M-15_, *bla*_CTX-M-14_ and *bla*_CTX-M-9_ are most frequent, especially in *E. coli*. The diversity of these enzymes and their resistance genes, carried by MGEs, plays a very important role, especially in their spread between different bacterial genera. ESBL detection is important for rapid and effective therapy. Currently, there are no recommended detection standards; there are many different methods, and it is up to each microbiology laboratory to decide on the most appropriate test. This further highlights the difficulties posed by these bacteria for researchers involved in the development of new antibiotics.

## Abbreviations

CLSI: Clinical and Laboratory Standards Institute
ESBL: extended-spectrum beta-lactamase
ESBL_A_: class A ESBL
ESBL_CARBA_: carbapenem-hydrolyzing ESBL
ESBL_M_: miscellaneous ESBL
EUCAST: European Committee on Antimicrobial Susceptibility Testing
3GCs: third-generation cephalosporins
IS: insertion sequence
MGEs: mobile genetic elements
WHO: World Health Organization
